# Genetic and Probiotic Characteristics of Urolithin A Producing *Enterococcus faecium* FUA027

**DOI:** 10.3390/foods12051021

**Published:** 2023-02-28

**Authors:** Mengjie Xia, Shuting Mu, Yaowei Fang, Xiaomeng Zhang, Guang Yang, Xiaoyue Hou, Fuxiang He, Yaling Zhao, Yichen Huang, Wei Zhang, Juan Shen, Shu Liu

**Affiliations:** 1 Co-Innovation Center of Jiangsu Marine Bio-Industry Technology, Jiangsu Ocean University, Lianyungang 222005, China; 2 Jiangsu Key Laboratory of Marine Bioresources and Environment/Jiangsu Key Laboratory of Marine Biotechnology, Jiangsu Ocean University, Lianyungang 222005, China; 3 School of Food Science and Engineering, Jiangsu Ocean University, Lianyungang 222005, China; 4 College of Food Science and Technology, Nanjing Agricultural University, Nanjing 210095, China

**Keywords:** *Enterococcus faecium*, urolithin A, safety assessment, genome, phenotypic assays

## Abstract

*Enterococcus faecium* FUA027 transforms ellagic acid (EA) to urolithin A (UA), which makes it a potential application in the preparation of UA by industrial fermentation. Here, the genetic and probiotic characteristics of *E. faecium* FUA027 were evaluated through whole-genome sequence analysis and phenotypic assays. The chromosome size of this strain was 2,718,096 bp, with a GC content of 38.27%. The whole-genome analysis revealed that the genome contained 18 antibiotic resistance genes and seven putative virulence factor genes. *E. faecium* FUA027 does not contain plasmids and mobile genetic elements (MGEs), and so the transmissibility of antibiotic resistance genes or putative virulence factors should not occur. Phenotypic testing further indicated that *E. faecium* FUA027 is sensitive to clinically relevant antibiotics. In addition, this bacterium exhibited no hemolytic activity, no biogenic amine production, and could significantly inhibit the growth of the quality control strain. In vitro viability was >60% in all simulated gastrointestinal environments, with good antioxidant activity. The study results suggest that *E. faecium* FUA027 has the potential to be used in industrial fermentation for the production of urolithin A.

## 1. Introduction

Ellagitannins (ETs), the metabolic precursor of urolithins, can be hydrolyzed to ellagic acid (EA), which is subsequently metabolized by gut microorganisms to urolithins [1]. Among all types of those urolithins, urolithin A (UA) exhibited several potentially positive bioactivities, such as restoring muscle function [2], and antiobesity [3], antioxidant [4], anti-inflammation, and anticancer activities [5]. An increasing amount of the literature has recently focused on the impact of the natural compound UA on health, disease, and aging [6]. Numerous studies have shown that different urolithin metabotypes (UMs) produce significantly different amounts and types of urolithins [7]. The gut microflora in more than 40% of middle-aged and elderly people cannot metabolize EA to UA [8]. Cortés et al. found that the percentage of the UM-A population declines when the intestinal flora changes with age [9]. Given the influence of intestinal flora on UA formation [10], screening strains responsible for metabolizing EA to produce UA is of interest.

Currently, little is known about the species of gut bacteria involved in EA conversion to UA. Strains found to metabolize EA to produce UA include *Bifidobacterium pseudocatenulatum* INIA P815 [11], *Streptococcus thermophilus* FUA329 [12], *Lactococcus garvieae* FUA009 [13], and *Enterococcus faecium* FUA027 [14]. *S. thermophilus* FUA329 was isolated from human milk. *L. garvieae* FUA009 and *E. faecium* FUA027 were screened from fecal samples. These bacteria have the potential to be developed as probiotics for the in vitro biotransformation of EA to produce UA, or for industrial fermentation to produce UA [15].

Our previous studies have proven that *E. faecium* FUA027, which was isolated from human fecal samples, metabolizes EA to UA by detecting UA from the fermentation broth of the strain through high-performance liquid chromatography (HPLC) and liquid chromatography tandem mass spectrometry (LC-MS/MS). The highest yield of UA produced by *E. faecium* FUA027 was 10.80 μM, thereby making this strain a promising candidate for development as a probiotic [14].

The safety and probiotic properties of the strain to be used as probiotics must be evaluated [16]. In this study, whole-genome sequence information analysis and phenotypic assays were used in combination to assess antibiotic resistance, metabolite toxicity, and survival under simulated gastrointestinal conditions. The safety of *E. faecium* FUA027 and its potential for use in the preparation of UA by industrial fermentation were confirmed.

## 2. Materials and Methods

### 2.1. Bacterial Strain and Growth Conditions

*E. faecium* FUA027 was preserved in the China General Microbiological Culture Collection Center (CGMCC) under the accession number CGMCC No. 24964. All FUA027 strains, unless otherwise noted, were cultivated in Anaerobe Basal Broth (ABB) medium and incubated under anaerobic conditions consisting of N_2_/H_2_/CO_2_ (80:10:10, *v*:*v*:*v*) at 37 °C for 24 h. *Staphylococcus aureus* ATCC 12600, *Escherichia coli* ATCC 25922, *Yeast* ATCC 24060, *Aspergillus niger* ATCC 6273, and *Lactobacillus plantarum* ATCC 4008 strains were used partly for inhibition experiments and partly as control strains in the experiments. *S. aureus* and *E. coli* were cultured at 37 °C in Luria–Bertani broth for 24 h. *Yeast* and *A. niger* were cultured on potato dextrose agar medium at 37 °C for 48 h. *L. plantarum* and *S. thermophilus* were cultivated in Man Rogosa Sharpe broth at 37 °C for 48 h.

### 2.2. Whole-Genome Sequencing

The genomic DNA was extracted from the *E. faecium* FUA027 culture grown in ABB by using a bacterial DNA extraction kit from Sangon, Shanghai, Co. Ltd. (Shanghai, China). For the DNA sample preparations, 1 µg DNA per sample was used as the input material. Sequencing libraries were created using the NEBNext^®^ Ultra™ DNA Library Prep Kit for Illumina (New England Biolabs, Ipswich, MA, USA) according to the manufacturer’s instructions. In brief, the DNA sample was sonicated to obtain 350-bp fragments. The DNA fragments were end-polished, A-tailed, and ligated with the full-length adaptor for Illumina sequencing with further PCR amplification. Finally, the AMPure XP system purified the PCR products, and the size distribution of the libraries was analyzed using the Agilent 2100 Bioanalyzer and quantified using real-time PCR. The whole genome of FUA027 was sequenced using the Nanopore PromethION platform and Illumina NovaSeq PE150 at the Beijing Novogene Bioinformatics Technology Co., Ltd. (Beijing, China).

### 2.3. Genome Assembly and Annotation

The trimmed data for the FUA027 genome were combined with PE150 and Nanopore data and assembled using SMRT Link v5.0.1 software (https://www.pacb.com/support/software-downloads/, accessed on 15 October 2022). The quality of the genome assembly was validated using QUAST ver. 5.0.2. The final assembly was annotated using the NCBI Prokaryotic Genome Annotation Pipeline (http://www.ncbi.nlm.nih.gov/genome/annotation_prok/, accessed on 15 October 2022) [17]. We used Gene Ontology (GO), the Kyoto Encyclopedia of Genes and Genomes (KEGG), Clusters of Orthologous Groups (COG), the Non-Redundant Protein Database, the Transporter Classification Database, and Swiss-Prot to predict gene function.

### 2.4. Strain Safety Assessment

#### 2.4.1. Identifying Safety-Related Genes from the FUA027 Genome

Bacterial virulence factors were identified by referring to the virulence factor database updated in 2019 (VFDB, http://www.mgc.ac.cn/VFs/, accessed on 11 October 2022) [18]. Protein sequences with >50% similarity in the extraction comparison results were identified as virulence genes. Antimicrobial resistance determinant identification was performed using the ABRicate program (https://github.com/tseemann/abricate, accessed on 11 October 2022) based on the ResFinder database (http://genomicepidemiology.org/, accessed on 11 October 2022) [19]. Antibiotic resistance genes of *E. faecium* FUA027 were identified using the comprehensive antibiotic resistance database (CARD, https://card.mcmaster.ca, accessed on 11 October 2022) [20].

#### 2.4.2. Antibiotic Susceptibility Testing

Susceptibility testing was performed through disk diffusion according to EUCAST recommendations [21]. The strain FUA027 was purified, inoculated into 20 mL of ABB liquid medium, and incubated anaerobically at 37 °C for 24 h. Bacterial colonies were counted, and the concentration of the bacterial solution was adjusted to 1.0 × 10^8^ CFU/mL. The bacterial solution was then added dropwise to a 20 mm agar plate. The FUA329 bacterial solution was evenly coated on the plate. Under aseptic conditions, antibiotic susceptibility papers were gently pressed onto the agar plates using forceps. While doing so, the spacing of each drug-sensitive tablet could not be <20 mm and the distance from the edge of the plate could not be <17 mm. The plates were sealed and continuously incubated at 37 °C for 14 h. The size of the inhibition circle was noted to determine the sensitivity of antibiotics.

#### 2.4.3. Hemolytic Activity Evaluation

The hemolytic activity was studied using the method described by Buxton. In short, *E. faecium* FUA027 was inoculated onto Columbia Blood Agar and incubated at 37 °C for 24 h [22]. *S. aureus* ATCC 12600 was used as a control strain.

#### 2.4.4. Nitrate Reductase and Amino Acid Decarboxylase Activity

The nitrate broth assay kit and amino acid decarboxylase assay kit obtained from Beijing Land Bridge Technology Co., Ltd. (Beijing, China). were used in the metabolic toxicity test. The test was performed following the manufacturer’s instructions.

Detection of nitrate reductase activity: Under aseptic conditions, single colonies of the test strain and the quality control strain *E. coli* ATCC 25922 isolated from the plate were inoculated in a nitrate broth assay ampoule by using an inoculating needle. The plate was incubated at 37 °C for 24 h. After incubation, nitrate reduction reagents A and B were added dropwise at 5:2 (*v*:*v*), and the results were observed immediately. Three parallel experiments were conducted for each sample [23].

Detection of amino acid decarboxylase activity: Under aseptic conditions, a single colony of the test strain was picked from the plate by using an inoculating needle and inoculated into the amino acid decarboxylase series ampoule as well as the amino acid decarboxylase control tube. Sterile liquid paraffin was added to cover the surface of the medium, and lysine, ornithine, and arginine ampoules were incubated at 37 °C for 24 h. After the phenylalanine ampoule was incubated for 24 h, 4–5 drops of 10% FeCl_3_ aqueous solution were added to the ampoules, and the results were observed within 2 min. Following the incubation of the tryptophan ampoules for 24 h, 2–3 drops of the Kovacs reagent were added to the ampoules and the results were observed immediately. Three parallel experiments were conducted for each sample.

### 2.5. Assessment of Probiotic Properties

#### 2.5.1. Probiotic-Associated Genes in the *E. faecium* FUA027 Genome

The Hidden Markov model (HMM) was used to find probiotic-associated genes in the genome as well as environmental tolerance-related genes [24]. Additionally, we searched for genes related to adhesion factors in the annotation results.

Putative genes involved in antimicrobial compound synthesis and secondary metabolism gene clusters in the *E. faecium* FUA027 genome were identified using AntiSMASH 6.0 (https://antismash.secondarymetabolites.org, accessed on 11 December 2022) [25] and BAGEL 4.0 (http://bagel4.molgenrug.nl/index.php, accessed on 11 December 2022) [26].

#### 2.5.2. Evaluation of Acid and Bile Salt Tolerance In Vitro

Referring to Pieniz et al.’s study, the survival of strains in a simulated gastrointestinal environment was measured using the viable plate count method [27]. The strain FUA027 was grown in ABB liquid medium at 37 °C for 24 h. Then, the culture was adjusted to an optical density (OD_600_) of 1.0 ± 0.05.

Separate preparation of ABB liquid medium of different pH values and containing different bile salt concentrations: test tubes containing 9 mL of ABB liquid medium were adjusted with HCl to attain different pH values (i.e., 2.0, 2.5, 3.0, 3.5, and 4.0). The ABB liquid medium was supplemented with bovine bile salt, thereby achieving final concentrations of 0.1%, 0.2%, 0.3%, 0.4%, and 0.5% (*w*/*v*), respectively. Then, 1 mL of inoculum was added to each tube, and the normal ABB liquid medium was used as a control. Sampling was performed at 0, 1, 2, and 3 h. The samples were diluted with ABB medium and then coated and incubated on the plates for 24 h, and viable colonies on a plate were counted. The survival rate was calculated using the following formula:

$$\mathrm{Survival}\;\mathrm{rate}\;\left(\%\right)=\left(N_{t}/N_{0}\right)\times 100\%$$

where $N_{t}$ (log CFU/mL) represents the number of viable bacteria after *t* hours of treatment, and $N_{0}$ (log CFU/mL) refers to the number of viable bacteria of *E. faecium* FUA027 before treatment.

#### 2.5.3. Evaluation of the Antioxidant Activity In Vitro

The FUA027 strain was cultured in ABB liquid medium at 37 °C for 18 h. The *E. faecium* FUA027 bacterial liquid was centrifuged (20 °C, 3000 rpm, 10 min), then discard supernatant intact cells of the strain were harvested. The cell pellet was washed twice with and suspended in 1 mL sterile distilled water [28]. The concentration of this suspension was adjusted to approximately 1.0 × 10^8^ CFU/mL. This was considered as a sample in the antioxidant test. Using antioxidant kits from Jiancheng Bioengineering Institute (Nanjing, China), in vitro antioxidant activities were measured including the measurement of the 2,2-diphenyl-1-picrylhydrazyl (DPPH) radical, hydroxyl radical, and superoxide anion scavenging activities [29].

#### 2.5.4. Hydrophobicity and Auto-Aggregation Tests

Hydrophobicity: The *E. faecium* FUA027 bacterial liquid was centrifuged (20 °C, 3000 rpm, 10 min), and the pellet was washed with and suspended in distilled water. The culture suspension was adjusted to an OD_600_ value of 0.5 ± 0.02 ($A_{0}$). Then, an equal volume of xylene solution was added to the bacterial suspension and vortexed for 20 s at 37 °C for 1 h. The absorbance of the supernatant at 600 nm ($A_{2}$) was determined. Three parallel tests were conducted [30]. The hydrophobic rate was calculated using the following formula:

$$\mathrm{Hydrophobic}\;\mathrm{rate}\;\left(\%\right)=\left(A_{0}/A_{2}\right)/A_{0}\times 100\%$$

Auto-aggregation: The FUA027 bacterial liquid was centrifuged (20 °C, 3000 rpm, 10 min) and washed with distilled water. Its OD_600_ value was adjusted to 0.5 ± 0.02 ($A_{0}$). The bacterial suspension was allowed to stand at 37 °C for 4 h, and the absorbance of the supernatant at 600 nm ($A_{2}$) was determined. Three parallel tests were conducted. The auto-aggregation rate was calculated using the following formula:

$$\mathrm{Auto}-\mathrm{aggregation}\;\mathrm{rate}\;\left(\%\right)=\left(A_{0}/A_{2}\right)/A_{0}\times 100\%$$

#### 2.5.5. Evaluation of Antibacterial Activity

A single colony of *E. faecium* FUA027 was picked, inoculated into ABB liquid medium, and cultured anaerobically at 37 °C for 24 h. Then, 10 mL of bacterial solution was mixed thoroughly with an equal volume of ethyl acetate extract, vortex shaken for 30 s, and transferred to a separatory funnel. This mixture was allowed to stand at room temperature for 5 min. After the solution was stratified, the upper organic phase was collected and evaporated in a rotary evaporation flask. The rotary evaporator was used to rotary evaporate the organic phase at 60 °C for 10–15 min to ensure the absence of a smell of ethyl acetate. Then, 2 mL ethyl acetate was added to dissolve the residue in the rotary steaming bottle, fully mixed, and filtered through a nylon syringe filter (pore size: 20 μm). The liquid was collected as the antibacterial solution [31]. The experimental group was the upper organic phase of *E. faecium* FUA027 after extraction with ethyl acetate (concentrated five times) and the lower aqueous phase of *E. faecium* FUA027 after extraction with ethyl acetate. ABB medium extracts and ethyl acetate were used as blank controls.

The Kirby–Bauer test for antibacterial effects: 100 μL of bacterial solution of *Staphylococcus aureus* ATCC 12600, *Escherichia coli* ATCC 25922, *Yeast* ATCC 24060, and *Aspergillus niger* ATCC 6273 were evenly applied to the plate, respectively. Then, four sterile filter papers of diameter 5 ± 0.5 mm were placed in each plate. A total of 10 μL of sample was added dropwise to each filter paper sheet and incubated for 12 h at 37 °C. Then, a vernier caliper was used to measure and record the diameter of the suppression ring. The inhibitory effect was evaluated on the basis of the inhibition circle diameter. Three independent tests were repeated [32].

## 3. Results and Discussion

### 3.1. Genome Properties

**Figure 1:**
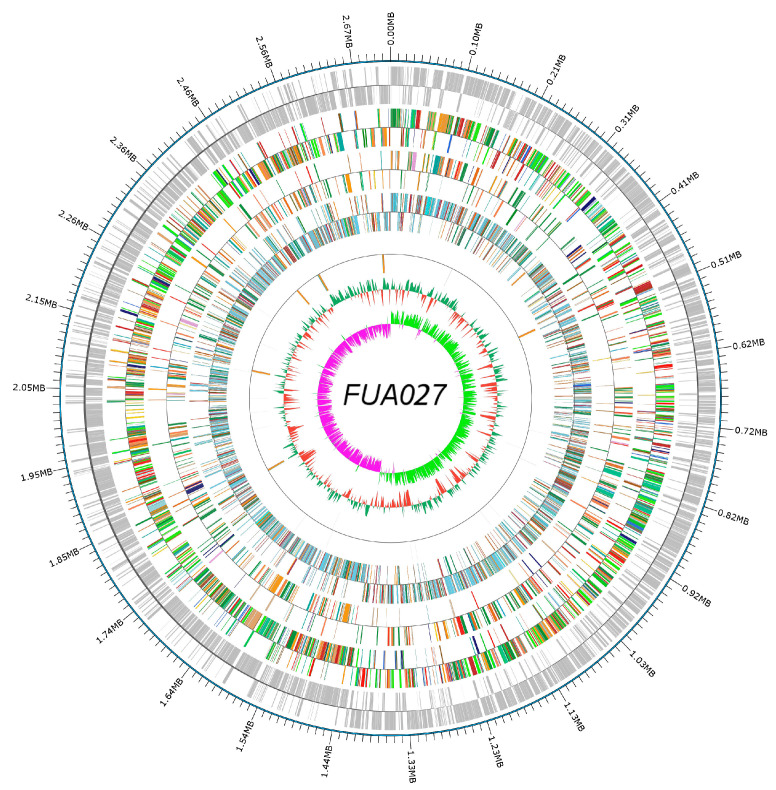
Whole genome map. The outermost circle is the genome sequence position coordinates, and from outside to center are the coding gene, gene function annotation results (COG, KEGG, GO database annotation results information), ncRNA, and genome GC content: the inward red part indicates that the GC content of the region is lower than the average GC content of the whole genome, and the outward green part is the opposite; and genomic GC skew value: the inward pink part indicates that the GC content of G in the region is lower than that of C; the outward light green part is the opposite.

The whole genome sequence of *E. faecium* FUA027 contained a single, circular 2,718,096-bp-long chromosome with an average GC content of 38.27% (Figure 1). The Glimmer program identified 2700 genes with an estimated coding ratio of 87.1%. Of them, 2617 were protein-coding genes and 83 were RNA genes. Among the 83 RNA genes, 17 genes coded for 5S, 16S, and 23S rRNAs; two genes coded for sRNAs; and 64 genes coded for tRNAs. (Table 1). The Plasmid Finder 2.0 tool did not find any plasmid sequences. The FUA027 genome sequence was submitted to NCBI under the accession number OM670243.

### 3.2. Evaluation Safety of E. faecium FUA027

#### 3.2.1. Identification of Antibiotic Resistance Gene

In the clinical setting, probiotic strains resistant to a particular antibiotic are typically associated with infection. Antibiotic resistance genes in the probiotic genome are not in themselves a safety issue, if the genes are not likely transferred to other strains. Instead, probiotics containing these genes could theoretically act as a source of antibiotic resistance genes for potentially pathogenic bacteria. Probiotics must also be tested for the presence of antibiotic resistance genes because studies have confirmed that these genes may be transferred in food and in the intestinal environment.

Enterococcus exhibits stronger natural resistance than other Gram-positive bacteria and acquires resistance genes through various mechanisms to produce multiple high-level drug-resistant strains [33]. Amino acid sequences of *E. faecium* FUA027 were compared with the drug resistance gene database CARD (https://card.mcmaster.ca/, accessed on 11 December 2022), and protein sequences with >50% similarity in the comparison results were extracted as antibiotic resistance genes. Eighteen antibiotic resistance genes were identified. A predictive analysis of drug resistance genes identified 10 types of aminoglycoside antibiotics, fluoroquinolones, lincosamides, and vancomycin. Probiotic *E. faecium* strain T-110 and non-pathogenic strain *E. faecium* NRRL B-2354 both contain a plasmid, according to Natarajan et al. [34]. Importantly, we used the MobileElementFinder tool to search for MGEs. As expected, the absence of MGEs was confirmed. Consequently, because *E. faecium* FUA027 has no plasmid and none of the antibiotic resistance genes associated with it are located on MGEs, these drug resistance properties cannot be transferred to other pathogenic bacteria through mobile elements, implying no occurrence of drug resistance transmission. Thus, this study from the genetic level confirms that *E. faecium* FUA027 is safe for the horizontal transfer of drug resistance. To corroborate the results of antibiotic resistance gene analyses, the antibiotic sensitivity test was conducted. Nevertheless, the presence of resistance genes did not exactly match the experimental results observed. According to the results, *E. faecium* FUA027 was resistant to nine antibiotic types (Table 2). In total, 27 antibiotics were detected. As shown in Table A1, *E. faecium* FUA027 was resistant to nine types of antibiotics. Combined with the results of the antibiotic susceptibility test in vitro, the antibiotic resistance genes in the genome were analyzed. *E. faecium* FUA027 was safe in terms of antibiotic resistance.

#### 3.2.2. Evaluation of Virulence Factor Genes and Toxin-Encoding Genes

**Figure 2:**
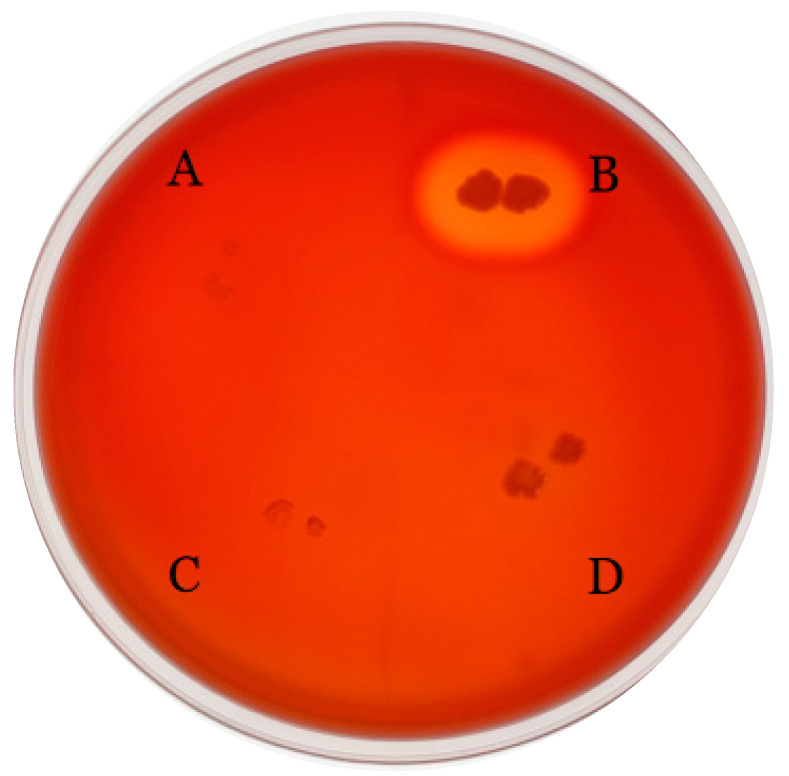
Blood agar plate test results. *S. thermophilus* (A), *S. aureus* (B), *S. thermophilus* FUA329, screening from human milk sample (C), and *E. faecium* FUA027 (D).

According to the gene function classification, virulence genes carried by enterococci mainly encode for proteins related to adherence, exotoxin, exoenzyme, immunomodulation, and biofilm [35]. The VFDB was used to identify virulence factor genes in *E. faecium* FUA027; however, most putative virulence factor genes had <60% similarity with VFDB [36]. In total, seven potential virulence factor genes were identified (Table 3). These genes may encode for proteins involved in adhesion, immunomodulation, exoenzyme, and biofilm. Genes encoding enterococcal hemolysin A (*hlyA*), cytolysin (*cyl*), aggregation substance (*as*), enterococcal surface protein, sex pheromones (*cob* and *ccf*), and serum resistance-associated gene (*sra*), which are well-known potential virulence factors, were missing in *E. faecium* FUA027. According to Deng’s study, among 110 probiotic *Enterococcus* spp. 35 (31.8%) enterococcal strains exhibited β-hemolytic characteristics. However, in our study, FUA027 exhibited γ-hemolysis on blood plates and no genes encoding *Hbl*, *Nhe*, or *cytotoxin K*, which are associated with hemolysis and toxin production, were found in the genome (Figure 2). These results thus confirmed that *E. faecium* FUA027 would be used in the preparation of UA by industrial fermentation.

#### 3.2.3. Biogenic Amine Production

The results of nitrate reductase activity revealed that *E. faecium* FUA027 did not contain nitrate reductase. No color change was observed in the tubes containing the test strains, and the color was red after the addition of trace zinc powder, indicating that the test group was negative. The tube containing the quality control strain *E. coli* ATCC 25922 was red and positive.

The amino acid decarboxylase activity of *E. faecium* FUA027 was preliminarily detected on the basis of the color change in the amino acid decarboxylase medium. With *E. faecium* FUA027, the color of the amino acid decarboxylase medium remained unchanged and yellow, indicating that no biogenic amines (BA) were produced in the medium by the strain. The experimental results revealed that FUA027 did not possess lysine, ornithine, arginine, tryptophan, or phenylalanine decarboxylase activities. The main source of BA in food is the microbial decarboxylation of amino acids. For example, the decarboxylation of tyrosine, ornithine, and lysine produces tyramine, putrescine, and cadaverine, respectively. BA accumulation in food has serious implications for food safety and human health [37]. Of the 129 enterococci strains of three different origins (food, veterinary, and human) screened by Sarantinopoulos et al., none produced histamine, cadaverine, or putrescine [38]. However, >90% of *E. faecium* strains isolated from cheese have been identified as tyramine producers. Some *E. faecium* strains from humans also produced putrescine [39]. *E. faecium* FUA027 was found to not produce BA, and thus we believe that this strain may be used safely in industrial fermentation.

### 3.3. Assessment of Probiotic Properties

#### 3.3.1. Acid and Bile Salt Tolerance In Vitro

**Figure 3:**
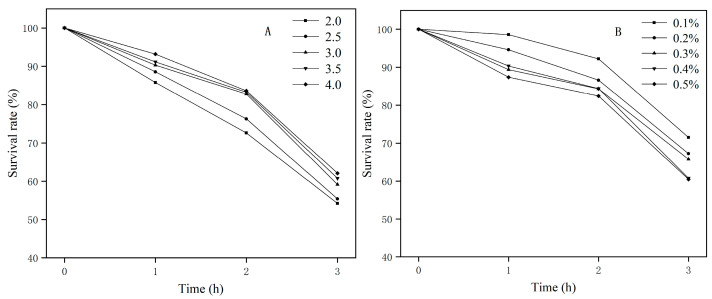
(**A**) Acid resistance test results; (**B**) bile salt resistance test results.

Normal human gastric juice pH is approximately 1–3, and normal human intestinal pH is approximately 6.8–7.0. The pH in the stomach can rise to 4–5 after food is consumed. Probiotics can only exert their probiotic role if they resist the inhibitory effects of gastric acid and pepsin on the intestine [40]. A gene encoding conjugated bile acid hydrolase (*cbh*) and three genes encoding bile acid sodium symporter family proteins were discovered in *E. faecium* FUA027; these genes may have contributed to bile salt resistance. F_0_F_1_-ATPase is considered the main pH regulator inside cells. Eight genes coding for the F_0_F_1_-ATP synthase subunit were identified in the FUA027 genome. Furthermore, a cation transporter gene, two (Na^+^/H^+^) antiporter genes, and a sodium ion transporter gene linked to pH regulation and ion homeostasis were discovered (Table A2). The survival rates of *E. faecium* FUA027 in the in vitro acid tolerance test at different pH values are shown in Figure 3A. The survival rate declined steadily as the pH value decreased. Studies have shown that strains with a survival rate of >60% are acid-resistant strains. The survival rate of *E. faecium* FUA027 in the in vitro acid tolerance test at pH 3.0 was >60% and that at pH 2.0 was >50%. Compared to acid-tolerant strains, *E. faecium* FUA027 was less acid-tolerant.

Another crucial sign for assessing the qualities of possible probiotics is the tolerance of strains to high bile salt concentrations in the human gastrointestine. Studies have shown that the small intestine contains approximately 0.3% of bile salts. In our study, the survival rate of the strain was higher than 67% at bile salt concentrations of 0.1%–0.3%. The strain survival rate was still >60.00% at bile salt concentrations of 0.4% and 0.5% (Figure 3B), which indicates that the strain has excellent bile salt resistance.

We identified a gene coding for conjugated bile acid hydrolase (*cbh*), two conjugated bile acid hydrolase genes (namely *nhaC* and *napA*), and ABC transporter genes potentially contributing to bile salt resistance in *E. faecium* FUA027. Eight genes coding for the F_0_F_1_-ATP synthase subunit (namely *atpB*, *atpE*, *atpF*, *atpH*, *atpA*, *atpG*, *atpD*, and *atpC*) were identified in the FUA027 genome. Therefore, we suggest that the in vitro results of acid and bile salt tolerance in *E. faecium* FUA027 are explained by these related genes in its genome.

#### 3.3.2. Antioxidant Ability In Vitro

Some probiotic metabolites can lessen the oxidative damage that causes aging and chronic diseases [41]. The results of the in vitro antioxidant ability of *E. faecium* FUA027 are presented in Table 4. The DPPH scavenging activity of the fermentation supernatant was as high as 57.62%, the superoxide anion scavenging capacity was 36.23%, and the clearance rate of hydroxyl radical was 30.12%. Polysaccharides, phosphonic acid, and peptidase, which are fundamental cell wall building blocks, are crucial for antioxidation. The extracellular metabolite structure is closely related to the antioxidant activity of the fermentation supernatant. In addition, the antioxidant activities of *L. plantarum* and *E. faecalis* were studied. The DPPH scavenging activity of *L. plantarum* was 62.78%, which was close to that of *E. faecium* FUA027. By contrast, the activity of *E. faecalis* was lower than that of *E. faecium* FUA027.

Ten genes associated with the oxidative stress response were found in the FUA027 genome; these genes could help the strain avoid damage by O^2−^ and H_2_O_2_^−^, such as peroxide-responsive repressor (*perR*), NADH peroxidase (*npr*), alkyl hydroperoxide reductase (*ahpC/F*), glutathione peroxidase (*gpx*), superoxide dismutase (*sodA*), thioredoxin reductase (*trxB*), and glutathione reductase (*gor*). Among them, *perR* regulates H_2_O_2_^−^ induced oxidative stress. In the presence of H_2_O_2_^−^ or with iron and manganese ion deficiencies, *perR* upregulates antioxidant enzymes such as *catA* and *ahpC/F* to scavenge H_2_O_2_^−^ and alkyl hydroperoxides (Table A2). The presence of these antioxidant genes indicated that *E. faecium* FUA027 has high antioxidant activity. Based on the results of genomic and phenotypic experimental analyses, we speculate that this may be due to the expression of antioxidant genes in the *E. faecium* FUA027 genome, such as catalase, glutathione peroxidase, and superoxide dismutation, which make FUA027 possess a good antioxidant capacity.

#### 3.3.3. Evaluation of Adhesion-Related Genes

Probiotics play a beneficial role by adhering to intestinal mucosa and epithelial cells. We searched for gene annotation data related to adhesion, colonization, mucin binding, flagella hook, and fibrinogen/fibronectin binding. Adhesion lipoprotein, s-ribosylhomocysteine lyasef (*luxS*), segregation and condensation protein B (*scpB*) were found in the *E. faecium* FUA027 genome (Table 5) [42].

Biofilms of lactic acid bacteria can colonize the intestine, thereby protecting strains in gastrointestinal transit, producing certain antimicrobial compounds, and stimulating the immune response. Auto-aggregation is a crucial property of biofilm formation, and hydrophobicity may assist in adhesion. Auto-aggregation and hydrophobicity are vital indicators of the ability of microbes to respond to bacterial gut colonization. FUA027 exhibited higher hydrophobicity and auto-aggregation than the commercial probiotic strain *Bifidobacterium longum* BB536). This demonstrates that *E. faecium* FUA027 can better colonize the intestinal tract, and thus exert its probiotic properties.

#### 3.3.4. Antibacterial Test of *E. faecium* FUA027 against Quality Control Strains

**Figure 4:**
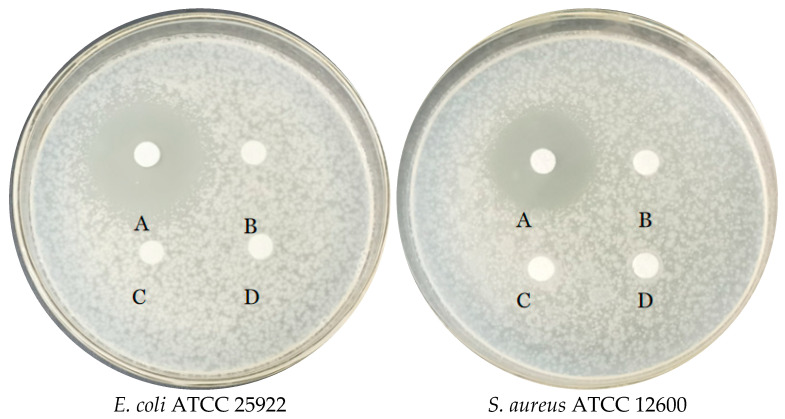

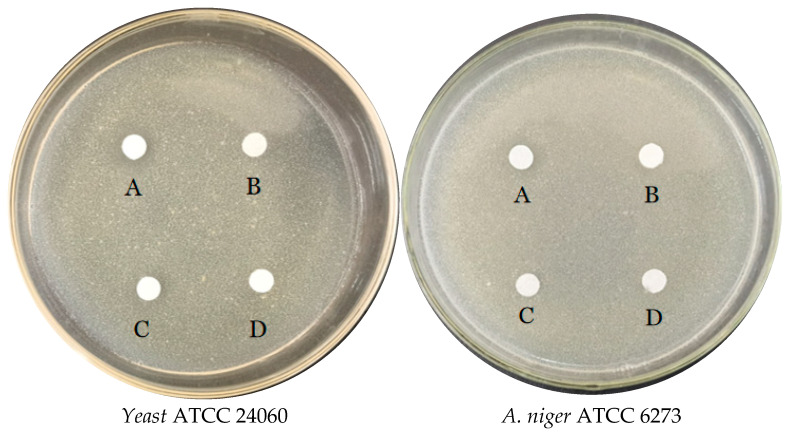
Antimicrobial activity of *E. faecium* FUA027 against quality control strains. The upper organic phase of *E. faecium* FUA027 fermentation liquid after extraction with ethyl acetate (concentrated five times) (A), the lower aqueous phase of *E. faecium* FUA027 after extraction with ethyl acetate (B), ABB medium extracts (C), and ethyl acetate (D).

In the in vitro experiment, the inhibitory ability of *E. faecium* FUA027 against four test strains was investigated. As shown in Figure 4, FUA027 exhibited significant inhibitory effects on *E. coli* ATCC 25922 and *S. aureus* ATCC 12600, with inhibition circle sizes of 26.24 ± 0.34 mm and 22.12 ± 0.26 mm, respectively. The inhibition circle sizes were 9.2 ± 0.52 mm and 8.74 ± 0.38 mm for *Yeast* ATCC 24060 and *A. niger* ATCC 6273, respectively. *E. faecium* FUA027 had a significantly better inhibitory effect on bacteria than on fungi. Antimicrobial activity is a crucial property of probiotics against gastrointestinal infections. *E. faecium* mainly exerts its bacteriostatic effect by secreting organic acids. Furthermore, bacteriocins, bacteriocin-like, and hydrogen peroxide secreted by *E. faecium* can inhibit intestinal pathogenic microorganisms to some extent. Many bacteriocin-producing *E. faecalis* strains have been reported. Rahmeh et al. explored how *E. faecium* S6 exerts its antimicrobial effect by producing enterotoxins and organic acids [43]. Valenzuela et al. isolated an *E. faecium* PE 2-2 strain from seafood that inhibited *S. aureus* and demonstrated that this strain carried the enterocin A structural gene [44]. Basanta et al. reported that *E. faecium* L50 isolated from a Spanish dry fermented sausage produces enterocin L50 (EntL50, EntL50A, and EntL50B), enterocin P, and enterocin Q and exhibits a broad antimicrobial spectrum [45]. Enterococins are often used as a preservative for meat and dairy products. The most widely used enterococins are enterocin A and enterocin B, belonging to class II bacteriocin. In our study, four biosynthetic gene clusters associated with T_3_PKS, a cyclic lactone autoinducer, were identified using AntiSMASH 5.0, and BAGEL 4.0 predicted a bacteriocin from the class sactipeptide in the *E. faecium* FUA027 genome. Sactipeptides (sulfur-to-alpha carbon thioether cross-linked peptides) are ribosomally synthesized and post-translationally modified peptides that exhibit antibacterial activity [46]. In conclusion, in vitro experiments supported the presence and activity of extracellularly secreted bacteriocins, as they significantly inhibit the growth of *E. coli* ATCC 25922 and *S. aureus* ATCC 12600.

## 4. Conclusions

In summary, we here described the whole-genome sequence of *E. faecium* FUA027. FUA027 has a 2,718,096-bp-long chromosome with an average GC content of 38.27%. Genomic screening revealed that FUA027 lacked key virulence factor genes and toxin-coding genes. Although 18 antibiotic resistance genes were screened from the strain, the strain has no plasmids or mobile elements and is therefore unlikely to undergo the acquisition and transfer of resistance genes. The safety of this strain was further confirmed through hemolysis tests, metabolic toxicity tests, and antibiotic resistance tests. The detection of antimicrobial gene clusters and adhesion- and stress-associated genes in the genome, along with the results of tolerance tests such as tolerance to acid and bile salt and in vitro antioxidant activity-related genes, revealed the probiotic properties of the strain. Genomic analysis combined with phenotypic studies confirmed the safety and probiotic properties of this strain as a potential probiotic candidate.

## Figures and Tables

**Table 1:** Characteristics of the *E. faecium* FUA027 genome.

Attribute	Value	% of Total
Total size, bp	2,718,096	100
Overall GC content	1,040,215	38.27
Total length of genes	2,367,573	87.1
Total genes	2700	100
Number of protein-coding genes	2617	96.93
Number of tRNA genes	64	2.37
Number of rRNA genes	17	0.63
Number of sRNA genes	2	0.07

**Table 2:** Putative antibiotic resistance genes identified in the genome of *E. faecium* FUA027.

Resistance Type	ARO_Name	Identity (%)	Gene Locus
Aminoglycoside antibiotic	*AAC*(*6’*)*-Ii*	100	GM_002230
	*AAC*(*3*)*-IIb*	59.1	GM_002310
	*ANT*(*4’*)*-Ib*	54.4	GM_001125
Lincosamide antibiotic	*msrC*	99.8	GM_002574
	*lsaA*	68.3	GM_000711
Fluoroquinolone antibiotic;	*efrA*	82.7	GM_002136
	*efrB*	81.7	GM_002135
	*efmA*	74.8	GM_000589
	*patB*	63.8	GM_002485
	*patA*	57.7	GM_002486
	*pmrA*	53	GM_001845
	*adeH*	60.4	GM_002244
	*arlR*	57.3	GM_001836
Diaminopyrimidine antibiotic	*dfrE*	62.6	GM_001700
Peptide antibiotic	*ugd*	58.7	GM_000959
Glycopeptide antibiotic	*vanRF*	51.5	GM_002680
Phosphonic acid antibiotic	*mdtG*	53.3	GM_000601
Rifamycin antibiotic	*Bado_rpoB_RIF*	52.8	GM_002629

**Table 3:** Putative virulence factors encoded in the *E. faecium* FUA027 genome.

Role	Virulence Factor	Related Genes	Identity (%)	Gene Locus
Adherence	Periplasmic solute binding protein	*efaA*	94.6	GM_000532
	Cell wall anchored protein	*SgrA*	77.8	GM_001311
	Collagen adhesin precursor	*Acm*	95.9	GM_002239
	Collagen adhesin protein	*Scm*	93	GM_002646
Immune modulation	Capsule	*capD*	56.4	GM_000949
Exoenzyme	Hyaluronidase	*EF0818*	60.8	GM_002331
Biofilm	Bopd	*EFAU085_00344*	97.6	GM_000438

**Table 4:** Antioxidant activity of *E. faecium* FUA027 in vitro.

Strain	DPPH	OH	O^2−^
	Fermentation Supernatant (%)	Fermentation Supernatant (%)	Fermentation Supernatant (%)
FUA027	57.62 ± 0.58	30.12 ± 0.76	36.23 ± 0.32
LP001	62.78 ± 0.72	28.54 ± 0.62	40.78 ± 0.24
FUA004	26.36 ± 0.54	9.56 ± 0.12	19.32 ± 0.58

**Table 5:** Cell adhesion-associated proteins in the *E. faecium* FUA027 genome.

Protein/Domain	Gene	Gene Locus
Segregation and condensation protein B	*scpB*	GM_001728
Segregation and condensation protein A	*scpA*	GM_001729
Sortase A	*strA*	GM_000664
S-ribosylhomocysteine lyase	*luxS*	GM_000502

## Data Availability

The data presented in this study are available on request from the corresponding author.

## Appendix A

**Table A1:** *E. faecium* FUA027 antibiotic resistance judgment criteria and test results.

Antibiotic	Standard for Judging Diameter of Inhibition Zone Diam (mm)			Drug Contents (µg)	Zone Diam (mm)	Antibacterial Effect
	R	I	S			
Amikacin	≤14	15~16	≥17	30	10 ± 0.2	R
Norfloxacin	≤12	13~16	≥17	10	19 ± 0.3	S
Ofloxacin	≤12	13~15	≥16	5	18 ± 0.4	S
Ciprofloxacin	≤15	16~20	≥21	5	15 ± 0.1	I
Levofloxacin	≤12	13~16	≥17	5	25 ± 0.3	S
Erythromycin	≤13	14~22	≥23	15	22 ± 0.1	I
Tetracycline	≤14	15~18	≥19	30	21 ± 0.2	S
Cefuroxime	≤14	15~17	≥18	30	30 ± 0.2	S
Cefazolin	≤14	-	≥15	30	22 ± 0.3	S
Cephalothin	≤14	15~17	≥18	30	27 ± 0.4	S
Cefotaxime	≤22	23~25	≥26	30	30 ± 0.1	S
Cefatriaxone	≤13	14~20	≥21	30	24 ± 0.4	S
Ceftazidime	≤14	15~17	≥18	30	18 ± 0.3	S
Piperacillin	≤28	-	≥29	100	21 ± 0.3	S
Ampicillin	≤16	18~24	≥25	10	22 ± 0.2	S
Oxacillin	≤17	-	≥25	1	14 ± 0.4	R
Penicillin G	≤28	-	≥29	10	22 ± 0.6	S
Aztreonam	≤15	16~21	≥22	30	0	R
Compound sulfamethoxazole	≤10	11~15	≥16	23.75	0	R
Nitrofurantoin	≤14	15~16	≥17	300	15 ± 0.6	I
Chloramphenicol	≤12	13~17	≥18	30	19 ± 0.3	S
Polymyxin B	≤11	12~14	≥15	300	0	R
Clindamycin	≤13	14~17	≥18	2	8 ± 0.4	R
Kanamycin	≤12	13~14	≥15	30	6 ± 0.4	R
Gentamicin	≤12	13~14	≥15	10	9 ± 0.6	R
Streptomycin	≤11	12~14	≥15	10	7 ± 0.7	R
Vancomycin	≤14	15~16	≥17	30	19 ± 0.3	S

**Table A2:** Stress-responsive proteins in the whole genome of *E. faecium* FUA027.

Type of Stress Response	Protein	Gene Locus
Acid stress response	F_0_F_1_-ATPase	GM_000855, GM_000856
		GM_000857, GM_000858
		GM_000859, GM_000860
		GM_000861, GM_000862
	Na^+^/H^+^ antiporter	GM_000704, GM_001361
Bile salts stress response	Conjugated bile acid hydrolase	GM_001069
	ABC transporter	GM_002271, GM_002452
		GM_000767, GM_002209
		GM_002135, GM_002210
		GM_002486, GM_002485
		GM_002270, GM_000917
		GM_000916, GM_002136
		GM_001223, GM_001224
Temperature stress response	Cold shock protein	GM_001318
Metal stress response	Divalent metal cation transporter	GM_000913
	Cadmium, zinc and cobalt-transporting ATPase	GM_000987
	Potassium/sodium uptake protein	GM_000822
Oxidative stress response	Alkyl hydroperoxide reductase	GM_001365, GM_001366
	Glutathione peroxidase	GM_000501
	Thioredoxin reductases	GM_000926
	Glutathione reductase	GM_002670
	Superoxide dismutase	GM_001908
	Peroxide-responsive repressor	GM_001511
	NADH peroxidase	GM_000518
	Superoxide dismutase	GM_001908
